# Comfrey: A Clinical Overview

**DOI:** 10.1002/ptr.4612

**Published:** 2012-02-23

**Authors:** Christiane Staiger

**Affiliations:** Merck Selbstmedikation GmbHRößlerstr. 96, 64293, Darmstadt Germany

**Keywords:** review, *Symphytum officinale* L., comfrey, Boraginaceae, clinical trial, non-interventional study, efficacy, safety

## Abstract

Comfrey has a centuries-old tradition as a medicinal plant. Today, multiple randomized controlled trials have demonstrated the efficacy and safety of comfrey preparations for the topical treatment of pain, inflammation and swelling of muscles and joints in degenerative arthritis, acute myalgia in the back, sprains, contusions and strains after sports injuries and accidents, also in children aged 3 or 4 and over. This paper provides information on clinical trials and non-interventional studies published on comfrey to date and further literature, substantiating the fact that topical comfrey preparations are a valuable therapy option for the treatment of painful muscle and joint complaints. Copyright © 2012 John Wiley & Sons, Ltd.

## INTRODUCTION

For centuries, comfrey has been used as a traditional medicinal plant for the treatment of painful muscle and joint complaints (Kothmann, 2003; Englert *et al*., 2005). Commonly found throughout Europe and parts of Asia, the plant also naturalized in North America, where it rapidly spread. Native Americans also recognized its healing powers and included comfrey in their therapeutic armamentarium (Hamel and Chiltoskey, 1975; Stammel, 1986). Comfrey has also been used in veterinary medicine (Rabinovich, 1981).

The German Commission E has assessed preparations containing *Symphytum officinale* L. positively for the treatment of blunt injuries (Kommission E, 1990a, 1990b). A European Scientific Cooperative on Phytotherapy Monograph is available for comfrey root (Symphyti radix; ESCOP, 2009). In addition, comfrey is described in the Hager Monographs (Staiger, 2009).

The constituents of comfrey root include 0.6–4.7% allantoin (Dennis *et al*., 1987); abundant mucilage polysaccharides (about 29%) composed of fructose and glucose units (Franz, 1969); phenolic acids such as rosmarinic acid (up to 0.2%), chlorogenic acid (0.012%) as well as caffeic acid (0.004%) and α-hydroxy caffeic acid (Andres, 1991; Grabias and Swiatek, 1998; Teuscher *et al*., 2009); glycopeptides and amino acids (Hiermann and Writzel, 1998); and triterpene saponins in the form of monodesmosidic and bidesmosidic glycosides based on the aglycones hederagenin (e.g. symphytoxide A), oleanolic acid (Aftab *et al*., 1996) and lithospermic acid (Wagner *et al*., 1970).

Comfrey root also consists of pyrrolizidine alkaloids with 1,2-unsaturated necine ring structures, almost entirely in the form of their N-oxides, the main ones being 7-acetylintermedine and 7-acetyllycopsamine together with smaller amounts of intermedine, lycopsamine and symphytine (Brauchli *et al*., 1982). The total amount of pyrrolizidine alkaloids given by different authors varies from 0.013% to 1.2% based on the analytical methods used (Tittel *et al*., 1979; Brauchli *et al*., 1982; Neidhardt, 1982; Stengl *et al*., 1982; Gracza *et al*., 1985; Vollmer *et al*., 1987; Mütterlein and Arnold, 1993).

The pyrrolizidine alkaloids echimidine and symlandine are not found in *S. officinale* L. and can be used as indicators of possible adulteration with other *Symphytum* species, such as *S*. × *uplandicum* or *S. asperum* (Mütterlein and Arnold, 1993). Nowadays, only pyrrolizidine-depleted or pyrrolizidine-free extracts are used in proprietary medicinal products. Special cultivars are also used (Schmidt, 2008).

The therapeutic properties of comfrey are based on its antiinflammatory and analgesic effects. Comfrey also stimulates granulation and tissue regeneration, and supports callus formation (Kommission E, 1990a, 1990b). However, the key activity-determining constituents of comfrey extracts and its molecular mechanisms of action have not been completely elucidated. Allantoin and rosmarinic acid are probably of central importance to its pharmacodynamic effects (Andres *et al*., 1989). No clinical-pharmacokinetic investigation results in humans have been published so far on the absorption, distribution and elimination of the constituents of comfrey extracts.

## *IN VITRO* AND *IN VIVO* DATA

Rosmarinic acid has been shown to possess antiinflammatory activity in various test systems. It inhibits the formation of malondialdehyde in human platelets (Gracza *et al*., 1985), prostaglandin synthesis, and carrageenan- and gelatine-induced erythrocyte aggregation (Gracza, 1987). In rat stomach preparations, a glycopeptide isolated from comfrey root dose-dependently inhibited the release of prostaglandins PGE2, PGI2, 12-HETE and arachidonic acid. An orally administered aqueous comfrey root extract inhibited carrageenan-induced rat paw oedema (Hiermann and Writzel, 1998).

In a study of the influence of a 60% ethanolic comfrey root extract on different elements of the human immune system, the extract was found to exert dose-dependent anticomplementary effects on the complement activation (van den Dungen, 1993). Antiinflammatory properties of a dry extract from comfrey root were also demonstrated in rats with induced paw oedema (Shipochliev *et al*., 1981; Mascolo *et al*., 1987; Hiermann and Writzel, 1998).

Wound-healing effects have been tested in 40% ethanolic comfrey root extracts and its high molecular weight (MW) fraction (> 1000 kD) in a test model of fibroblasts in a collagen matrix. Both inhibited shrinkage of the collagen matrix (van den Dungen *et al*., 1990; van den Dungen, 1993).

## COMFREY HERB AND LEAVES

Besides the comfrey roots, all the parts of the plant that grow above ground (Symphyti herba) or the leaves (Symphyti folium) are also utilized for medical purposes (Schmidt, 2006). The indications for which randomized clinical trials of ointments containing these kinds of extracts have been conducted include wound healing, myalgia and acute ankle joint distortions.

### Wound healing

A topically applied preparation containing 10% active ingredient from the aerial parts of comfrey (*Symphytum* × *uplandicum* Nyman, Traumaplant®) was examined for its wound-healing effects (Barna *et al*., 2007). The randomized, double-blind clinical trial included 278 patients (verum: *n* = 137) with fresh abrasions. The subjects included 64 patients of up to 20 yr of age (verum *n* = 29, reference product *n* = 35). An otherwise identical low-dose preparation (1% active ingredient; *n* = 141) was used as a reference.

After 2 to 3 days, a significantly and clinically relevantly faster initial reduction in wound size of 49 ± 19% versus 29 ± 13% per day in favour of verum (*p* < 5 × 10^−21^) was found. From linear regression time to complete healing was determined to be 2.97 days faster with verum than with the reference (4.08 vs. 7.05 days, *p* = 7.4 × 10^−45^ in the t-test comparison of regression lines). The physicians rated efficacy as good to very good in 93.4% of cases, as compared with 61.7% in the group treated with the reference product (*p* = 2 × 10^−11^). A subgroup analysis found no significant influence of abrasion area, gender or age on healing effects, albeit that a tendency towards better effects with increasing age was observed. No adverse effects or problems with drug tolerability were observed.

### Myalgia

The same topical *Symphytum* product was tested for its effectiveness and tolerability in the treatment of patients with myalgia (n = 104; Kucera *et al*., 2005). Again, an otherwise identical low-dose preparation (1% active ingredient; *n* = 111) was used as a reference. This double-blind, reference-controlled, randomized, multicentre trial included 215 patients with pain in the lower and upper back. The primary efficacy parameter was pain in motion, assessed with the aid of a visual analogue scale. Secondary efficacy parameters included pain at rest, pain on palpation and functional impairment. With high concentrations of the treatment product, amelioration of pain on active motion (*p* < 5 × 10^−9^), pain at rest (*p* < 0.001) and pain on palpation (*p* = 5 × 10^−5^) was significantly more pronounced than with the reference product and was clinically highly relevant. A number needed to treat of 3.2 was calculated from the study results. Global efficacy was significantly better (*p* = 1 × 10^−8^) and onset of effect was faster (*p* = 4 × 10^−7^) with the high-concentration product. Tolerability of the highly concentrated study product was reported good to excellent in all patients.

### Distortion

A randomized, multicentre, double-blind study including 203 patients confirmed the efficacy of the same comfrey herb preparation (10% active ingredient of a 2.5:1 aqueous-ethanolic pressed concentrate of freshly harvested, cultivated comfrey herb [*Symphytum* x *uplandicum* Nyman], corresponding to 25 g of fresh herb per 100 g of cream) in acute ankle joint distortions, particularly with regard to pain reduction (Kucera *et al*., 2004). Efficacy and tolerability were compared with a reference product containing 1% of the active ingredient (corresponding to 2.5 g of fresh comfrey herb in 100 g of cream). The reduction of symptom scores for pain when moving, pain at rest and functional restrictions under verum was significant and clinically relevant (*p* < 0.001) on days 3, 4 and 7. Compared with the reference product, reduction of swelling on days 3 and 4 was equally significant (*p* < 0.01). One comment on the trial emphasized that using a comparator containing very little of the active ingredient instead of a placebo might be a good approach for clinical trials with herbals, when blinding is difficult (Schulz, 2005a).

## OTHER CLINICAL STUDIES AND POST-MARKETING SURVEILLANCE

Several open and post-marketing surveillance studies have also been published. A recent study also included children 4 to 12 yr of age. However, some of the studies are uncontrolled and older than 15 yr.

### Children

In an open observational study the above mentioned topical cream was tested in 196 children from the ages of 4 to 12 yr with respect to the paediatric treatment of acute blunt traumata (contusions, strains and distortions; Grünwald *et al*., 2010). The symptoms pain on palpitation, pain in motion, functional impairment, oedema and haematoma were included in the evaluation. The average duration of the administration of the trial sample was 7.6 ± 1.1 days. The remission rates for the symptoms were 86.3% (pain on palpitation), 86.7% (pain in motion), 89.7% (functional impairment), 94% (oedema), 87.6% (haematoma), and 90.1% (impairment of general condition). No adverse drug reactions occurred.

### Muscles and joints

In another open uncontrolled study, the efficacy of the same registered drug containing comfrey herb extract was tested on 105 patients suffering from locomotor system symptoms (Kucera *et al*., 2000). The cream was applied twice daily. Functional disturbances and pain completely resolved in 57 of the 105 patients. A further 24 patients achieved normalization of function with continued moderately severe pain. Moderate improvement occurred in 21 patients, and three patients reported no improvement in their condition. Muscle pain proved most amenable to treatment with the cream, swelling and overstrain also responded well. The treatment was less efficacious against pain accompanying osteoporosis.

In a study involving 30 patients, the effect of the same preparation was assessed in the treatment of acute supraspinatus tendon syndrome (Mayer, 1993). The ointment was combined in the form of a supplementary percutaneous therapy with local infiltration therapy during the 3-week control period. Compared with the control group, the ointment containing comfrey extracts proved to have a significantly superior effect with regard to the reduction of pain and the associated functional restrictions.

The ointment containing comfrey herb extract was also used to treat 22 additional patients suffering from acute contusions and distortions of the knee joint, and the following clinical symptoms were measured afterwards: swelling of the joint, active and passive pain when moving and local pain when resting (Mayer, 1992). Application of the ointment resulted in a significant alleviation of pain after only 4 days of treatment. All patients were completely pain-free after 10–14 days.

In a 2-week controlled study, the effect of the same ointment was compared with conventional cryotherapy in treating patients with acute ankle joint distortion (Mayer, 1991). Test criteria in this study also included pain at rest, pain when moving and swelling. Symptoms improved significantly quicker during comfrey treatment than during cryotherapy. Application of the ointment containing comfrey proved to be more compliance friendly than cryotherapy.

In another study involving patients with acute contusions and strain traumas of the knee joint, efficacy was proven to be good or even very good and a significant therapeutic impact on the damaged joint became apparent (Hess, 1991). Forty patients suffering from knee joint injuries, sprains and bruises were treated with ointment containing comfrey extract, achieving a significant reduction of pain (pain at rest and on movement) and swelling. The mobility of the affected joint increased significantly. Treatment took place over a period of 8 days and had a good to very good effect on 85% of the patients.

### Wounds

A further study examined the effect of an ointment containing *Symphytum peregrinum* extract in comparison with an active ingredient-free ointment base, i.e. an active ingredient-free polyacrylamide gel, in 10 patients with experimentally produced flat open wounds with the stratum basale intact (Niedner, 1989). The healing time when using ointment containing comfrey extract was significantly shorter than when applying comparative preparations. The difference with regard to the active ingredient-free ointment base was statistically significant.

## COMFREY ROOT: RANDOMIZED CLINICAL TRIALS

The medicinal use of preparations from the underground parts of the plants (Symphyti radix) is well established. Relevant medicinal products are now marketed in more than 10 countries and the present licences include the topical treatment of pain, inflammation and swelling of muscles and joints in the case of degenerative arthritis, acute myalgia in the back, sprains, contusions and strains after sports injuries and accidents, also in children aged 3 and over. Corresponding randomized clinical trials and non-interventional studies studied the efficacy of comfrey root extract ointment for treatment of various muscle and joint complaints (Staiger, 2005, 2007).

### Back pain

A double-blind, placebo-controlled, multicentre, randomized clinical trial with parallel group design was conducted over a period of 5 days (Giannetti *et al*., 2010). One-hundred and twenty patients with acute upper or lower back pain were treated three times a day, 4 g per application. They used either a verum cream containing comfrey root fluid extract (1:2, 35.0 g, extraction solvent ethanol 60% (v/v), less than 0.35 ppm of pyrrolizidine alkaloids, Kytta-Salbe®f) or a corresponding placebo. The trial included four visits and was performed at the German Sport University in Cologne (Deutsche Sporthochschule) and three additional ambulatory centres for orthopaedics and sports medicine.

The primary efficacy variable was the area under the curve (AUC) of the Visual Analogue Scale (VAS) on active standardized movement values at visits 1 to 4. The pain intensity on VAS was assessed at performance of standardized, muscle group specific tests. The secondary objectives were back pain at rest using assessment by patient on VAS, pressure algometry (pain–time curve; AUC over 5 days), global assessment of efficacy by the patient and the investigator, intake of analgesic medication and functional impairment measured with the Oswestry Disability Index.

There was a significant treatment difference between comfrey root extract and placebo regarding the primary and secondary variables. The pain intensity on active standardized movement decreased on average (median) approximately 95.2% in the comfrey extract group (104.8–12.7 mm; mean VAS sum) and 37.8% in the placebo group (100.0–56.5 mm; mean VAS sum) (*p* < 0,001). Compared with placebo, superiority of the verum treatment was significant with regard to secondary efficacy variables (each *p* < 0.001). Both the AUC of the reported back pain at rest, the AUC of the pressure algometry in the trigger point as well as the global assessment of the efficacy by the patients and the investigators showed a clinically relevant effect in reducing acute back pain. For the first time, a fast-acting effect of the ointment (1 h) was also observed. After 1 h the pain intensity had already decreased about 33.0% in the comfrey group (104.8 to 60.4 mm; mean VAS sum) and 12.0% in the placebo group (100.00 to 86.5; mean VAS sum) indicating an early onset of the treatment effect. A total of seven patients experienced adverse events in the course of the clinical trial, four in the comfrey extract group and three in the placebo group. Eczema, cold, nausea and rhinitis occurred in the verum group, headache (*n* = 2) and pruritus in the placebo group. All adverse events were of mild severity.

One comment on the trial asked for more data in patients with different sorts of other back pain but admits that the results are relevant and topical treatment is increasingly considered as a serious treatment option (Rannou, 2010).

### Painful osteoarthritis

The same cream was investigated in a randomized, double-blind trial including 220 patients suffering from painful osteoarthritis of the knee (Grube *et al*., 2007). All patients met the criteria of the American College of Rheumatology and received 2 g of either the active or a corresponding placebo cream three times a day for 21 days.

Pain, functional impairment and stiffness are the most important symptoms patients seek to relieve. Therefore, the primary target variable was the VAS sum score of pain at rest and pain on movement. A secondary target variable was the Western Ontario and McMaster Universities (WOMAC) score. During the course of the study, the total score of the primary target variable decreased by 51.6 mm (54.7%) in the verum group and 10.1 mm (10.7%) in the placebo group, a significant difference of 41.5 mm (44.0%) between groups (*p* < 0.001). The secondary target criterion reduced by 60.4 mm (58.0%) in the verum group and 14.7 mm (14.1%) in the placebo group, the difference of 45.7 mm (43.9%) again being significant (*p* < 0.001).

Superiority of improvement in the verum group was also evident with respect to four explorative secondary parameters: SF-36 (quality of life), angle measurement (mobility of the knee), CGI (clinical global impression) and global assessment of efficacy by physicians and patients (*p* < 0.001 for each parameter). A total of 22 AEs occurred in 22 patients (7 in the active therapy group, 15 in the placebo group). No adverse drug reaction was reported in the active therapy group.

One comment on the trial mentioned the difficulties that are usually associated with the production of placebos for herbal drugs. It emphasized that due to the low inherent smell of the extract and the same perfume used in both placebo and verum, a very good blinding could be achieved for this preparation (Schulz, 2007). Another comment found the trial to be well conducted and in accordance with the GCP-ICH guidelines (Chrubasik, 2007).

### Blunt injuries

In a double-blind, multicentre, randomized, placebo-controlled, group comparison clinical trial on patients suffering from ankle distortion, the percutaneous efficacy of the same cream of comfrey extract was confirmed decisively (Koll *et al*., 2000, 2004). The mean age of the 142 patients was 31.8 yr; 78.9% were male. The inclusion criterion was an uncomplicated, acute unilateral ankle distortion that had occurred no longer than 6 h previously. The duration of treatment was 8 days. Local treatment of the afflicted ankle was performed with ca. 2 g (a 6-cm strand of cream) of either verum or placebo.

The primary variable, tenderness of the ankle joint, was measured by pressure algometry, meaning the difference in tolerated pressure between injured and healthy ankles. Under active treatment, no adverse drug reactions were reported. During the course of treatment, pain regressed significantly more in the comfrey extract group than in the placebo group (*p* < 0.0001) and at the final assessment the reductions in tenderness compared with initial values were 2.44 kp/cm^2^ in the verum group compared with only 0.95 kp/cm^2^ in the placebo group. Compared with placebo, superiority of the verum treatment was significant with regard to reduction in pressure pain (tonometric method, *p* < 0.0001), ankle oedema (figure-of-eight method, *p* = 0.0001), ankle mobility (dorsiflexion, *p* = 0.002; plantar flexion, *p* = 0.0116) and global efficacy (*p* < 0.0001). A comment valued the trial as a well-executed and designed randomized clinical trial (RCT) with clearly shown beneficial effects (De Lange-de Klerk, 2005).

### Verum-controlled versus Diclofenac

In a single-blind, controlled, randomized, parallel groups, multicentre and confirmatory clinical trial outpatients with acute unilateral ankle sprains (*n* = 164) received either a 6-cm-long ointment layer of the aforementioned comfrey root extract cream (*n* = 82) or of diclofenac gel containing 1.16 g of diclofenac diethylamine salt (*n* = 82; Predel *et al*., 2005). They applied the cream for 7 days, four times a day. The primary efficacy variable was pain arising from pressure on the injured area, measured with a calibrated caliper (algometer) on days 0, 4 and 7 and evaluated by the area under the curve (AUC) of the pain–time curve. Secondary variables were the circumference of the joint (swelling, figure-of-eight method), the individual spontaneous pain sensation at rest and at movement according to a VAS, the global efficacy evaluation, the global assessment of tolerability and further variables. It was confirmatorily shown that comfrey extract is non-inferior to diclofenac. The 95% confidence interval for the AUC (comfrey extract minus diclofenac gel) was 19.08 to 103.09 h*N/cm^2^ and completely above the margin of non-inferiority. After 7 days of treatment a mean relative reduction in VAS at rest of 92% was found in the comfrey cream group. The corresponding reduction in the diclofenac group was 85%. The mean relative reductions in VAS in motion were 83.2% for comfrey extract and 72.4% for diclofenac. Ankle swelling decreased by 79.5% in the comfrey root and 69.4% in the diclofenac group. The pain on pressure measured with an algometer was reduced by 80.6% in the comfrey root, but only by 74.7% in the diclofenac group.

A re-evaluation of the trial data in accordance with Committee for Proprietary Medicinal Products guidelines (CPMP, 2000) even revealed superiority of the herbal medicine in several parameters (D'Anchise *et al*., 2007). In the primary variable the comfrey root extract cream showed a statistically significant superiority above the diclofenac gel (*p* = 0.0012). On day 4, a statistically significant reduction of the pain on pressure (*p* = 0.0449), and on day 4 (*p* = 0.0368) and day 7 (*p* = 0.0074) a statistically significant reduction of the pain on movement was recorded. Further, the physicians (*p* = 0.0130) as well as the patients (*p* = 0.0111) rated the global efficacy of the comfrey preparation significantly higher than the efficacy of the diclofenac gel.

Comments on the trial appreciated the proof of efficacy compared with the chemical comparator (Schulz, 2005b) and referred to the observer-blind design as the best possible in cases where a double-blind design cannot be performed (Chrubasik, 2006).

### Other clinical trials

In an earlier 4-week pilot study, 41 patients with different forms of musculoskeletal rheumatism (mainly epicondylitis, tendovaginitis and periarthritis) were treated topically with the same cream as above (*n* = 20) or with placebo (*n* = 21) (Petersen *et al*., 1993). Efficacy was assessed using several pain parameters: tenderness when pressure applied, pain at rest and during exercise. With respect to ‘tenderness when pressure applied’, the ointment proved superior to placebo in patients with epicondylitis and tendovaginitis, but not in patients with periarthritis.

The effects of dermatological preparations containing 5% or 10% of a comfrey root extract (2:7, 50% ethanol) on the process of healing of experimentally induced UV-B erythema were studied in 29 volunteers in a controlled pharmacological trial (Andres *et al*., 1989; Andres, 1991). The antiinflammatory potency of the extract was found to be equal to or greater than that of diclofenac. A positive correlation could be demonstrated between efficacy and the concentration of α-hydroxy caffeic acid in the extract, but not for allantoin.

## POST-MARKETING SURVEILLANCE

The results of the non-interventional studies are in line with the results of the aforementioned clinical trials. In particular, data for children aged 3 to 12 yr is available.

### Children

In a non-interventional study of a comfrey root extract cream containing 35% of a comfrey root extract (1:2, ethanol 60% (v/v)) the tolerability and efficacy were examined in 306 children aged 3 to 12 yr (Staiger and Wegener, 2008). The preparation was used to treat a variety of conditions such as contusions (61.4%), strains (14.1%), distortions (30.4%) and other indications (6.9%). The ointment was applied to most of the children three times daily (57.8%), four times daily (26.1%) or twice a day (13.4%). Thereby the physicians administered mostly the same dosages as for adults and children aged 12 yr and older. In the overall score of the findings pain on palpation, restriction of movement and haematoma manifestation (minimum 3, maximum 15) a notable improvement in the clinical result became clear: the initial value of 10.61 fell by 6.18 points or by 58.3%. Clear remission or improvement was revealed in every individual finding. For all clinical symptoms, an improvement of over 50% could be calculated. The most marked reduction was in pain at rest (62.6%), restriction of movement (62.0%) and pain sensitivity (61.4%).

### Comfrey cream

In a post-marketing surveillance study, 163 patients with a mean age of 45.3 yr applied the same comfrey root extract cream for several conditions, the most frequent being contusions (33.1%), painful joint complaints (27.6%), sprains (26.4%) and painful muscle complaints (23.3%; Tschaikin, 2004). Most patients applied the preparation two (38%) or three (48.5%) times daily. The median duration of treatment was 11.5 days. During the observation period symptoms of pain at rest and during the night, pain during motion, tenderness when pressure applied, impaired mobility, painful muscle complaints and swellings improved markedly. Morning joint stiffness decreased by 94% from 17 min initially to 1 min. The use of non-steroidal antiinflammatory drugs (NSAIDs) was reduced or discontinued by 13.5% of patients. The physicians assessed global efficacy as excellent in 38.7% of cases and good in 54.6%.

### Comfrey paste

In a simultaneous surveillance study, 162 patients applied a similar preparation, a paste containing 30% of the above mentioned fluid extract of comfrey roots (Pabst and Ottersbach, 2004). They also treated a variety of conditions such as painful joint complaints (34%), contusions (26.5%) or painful muscle complaints (21.6%). Most patients applied the preparation once (23.5%) or twice (52.5%) daily. The median duration of treatment was 11.8 days. Again, symptoms of pain at rest and pain during movement, impaired mobility, swelling and painful muscle complaints improved markedly during the observation period. Morning stiffness of investigated joints decreased by 90% from 20 min initially to 2 min. The use of NSAIDs was reduced or discontinued by 21% of patients. Global efficacy was assessed by the physicians as excellent in 65.4% of cases and good in 32.7%.

### Combination with methyl nicotinate

A cream consisting of a combination of 35% of comfrey root fluid extract and 1.2 % methyl nicotinate is available in Germany, Luxembourg and Switzerland. A further simultaneous non-interventional study of this preparation included 162 patients (Klingenburg, 2004). The mean age of the patients was 49.7 yr, the mean duration of treatment 12.3 days. Pain at rest and during the night was reduced by 45%, pain during motion by 47%, tenderness when pressure applied by 47%, painful muscle complaints by 48%, and impaired mobility improved by 46%. In the course of the study, four patients with seven non-serious, resolved adverse events, namely skin reactions such as redness or itching, were recorded.

### Other data

A total data analysis of the previous three post-marketing surveillance studies is also available (Koll and Klingenburg, 2002). The findings are in line with the aforementioned results. With regard to all 492 patients, pain at rest, pain in movement, and tenderness when pressure applied improved, decreasing by 45–47% on average.

The effects of dermatological preparations containing 5% or 10% of a comfrey root extract (2:7, 50% ethanol) on the process of healing of experimentally induced UV-B erythema were studied in 29 volunteers in a controlled trial. The antiinflammatory potency of the extract was found to be equal to or greater than that of diclofenac. A positive correlation could be demonstrated between efficacy and the concentration of a caffeic acid derivative in the extract, but not for allantoin (Andres *et al*., 1989; Andres, 1991).

Comfrey root has also been used for knee joint injuries and non-active gonarthrosis, as well as in the treatment of tendinitis syndrome, insect bites, mastitis, fractures, skin inflammation, multiple abscesses of sweat glands, gangrenous ecthymas, furuncles, dicubital ulcers and chronic varicose ulceration, as prior studies and individual case reports reflect (Häberle, 1952; Briel, 1953; Ziolkowski *et al*., 1957; Korte and Rapp, 1958; Büzberger, 1960; Prinzing, 1960; Deister, 1963; Awang, 1987; Koehler and Franz, 1987; Kothmann, 2003; Barnes *et al*., 2007).

## SAFETY

With regard to safety, the absence of genotoxic effects was demonstrated in the bacterial reverse mutation assay (Ames test) for a pyrrolizidine alkaloids (PA)-free comfrey root liquid extract (Benedek *et al*., 2010). The extract was investigated for its ability to induce gene mutations in *Salmonella typhimurium* strains TA 98, TA 100, TA 102, TA 1535 and TA 1537 with and without metabolic activation using the mammalian microsomal fraction S9 mix. Reference mutagens were used to check the validity of the experiments. The comfrey root extract showed no biologically relevant increases in revertant colony numbers of any of the five tester strains, neither in the presence nor in the absence of metabolic activation. In conclusion, the fluid extract was not mutagenic in the bacterial reverse mutation assay.

Literature on comfrey often concentrates on PAs, recommending a restriction of the duration of treatment, also with externally applied comfrey preparations. However, in Germany, the restriction limiting application to 4–6 weeks/yr applies only to preparations containing more than 10 µg, but less than 100 µg pyrrolizidine alkaloids (daily allowance; Bundesgesundheitsamt, 1992). Fully licenced medicinal products available today contain depleted or PA-free extracts. The application results in far below the daily allowance of 10 µg. As a consequence there are no restrictions in Germany on these products as regards the duration of treatment (Bundesgesundheitsamt, 1992).

## DISCUSSION

Comfrey has a long tradition as a medicinal plant. In general, the effects of comfrey extracts can be described as pain relieving, antiinflammatory and callus formation promoting. To date, the activity-determining constituents and mechanisms of action of the medicinal plant are only partly known. However, in accordance with the modern approach of evidence-based medicine, comfrey extract creams have demonstrated their efficacy and tolerability in a number of muscle and joint injuries, such as acute myalgia in the back area, and in blunt injuries. Comfrey herb has also been shown to be efficacious in wound healing. Comfrey root has also proven to be efficacious in activated osteoarthritis, and equivalent or more efficacious in distortions compared with topical diclofenac. Although for each indication and licenced product only one modern randomized clinical trial is available so far, they all point to the pain-relieving effect in muscle and joint complaints. It could therefore be promising to investigate topical comfrey preparations in further indications related to muscle or joint pain, for instance chronic forms of back pain.

## CONCLUSION

In the 17th century, Nicholas Culpeper (1616–1654) mentioned comfrey in his enlarged version of *The English Physitian* (Culpeper, 1656). He stated: ‘It is said to be so powerful to consolidate and knit together (…) and a Syrup made thereof is very effectual for all those (…) outward Wounds and Sores in the Fleshy or Sinewy part of the Body whatsoever’. He recommended comfrey among many other complaints for ‘Inward Wounds & Bruises, Wounds, Ruptures, broken Bones, Inflamation, Gout, and Pained Joynts.’

Today, this historical statement is widely supported by modern clinical data. Several recent randomized clinical trials substantiate the efficacy of topical comfrey preparations in the treatment of pain, inflammation and swelling of muscles and joints in the case of degenerative arthritis, acute myalgia in the back, sprains, contusions and strains after sports injuries and accidents, also in children aged 3 and over.

## Conflict of Interest

Christiane Staiger is an employee of Merck Selbstmedikation GmbH. A few products in the company's portfolio contain comfrey root extract.
